# Antioxidant and Immunomodulatory Activities of Polysaccharides from Fermented Wheat Products of *Grifola frondosa*: *In Vitro* Methods

**DOI:** 10.1155/2023/3820276

**Published:** 2023-08-09

**Authors:** Xiaoqing Xu, Ying Liu, Chunli Pan, Shaoliang Han, Lan Ma, Yu Qiao, Bo Shi, Qing Peng

**Affiliations:** ^1^State Key Laboratory of Feed Microorganism Engineering, Beijing Dabeinong Science and Technology Group Co., Ltd., Beijing, China; ^2^Institute of Feed Research, Chinese Academy of Agricultural Sciences, Beijing, China

## Abstract

Despite the well-known health benefits of *Grifola frondosa*, there is a lack of understanding regarding the potential antioxidant and immunomodulatory properties of different varieties when fermented with wheat grains. We aimed to explore the potential of *G. frondosa*-fermented wheat flour as a functional food. Three varieties of *G. frondosa* (GFA, GFB, and GFC) were fermented with wheat grains for solid-state fermentation. Polysaccharides were extracted and analyzed for total sugar content, monosaccharide composition, Mw profile, antioxidant activity, cytotoxicity, and immunomodulatory properties. Results were evaluated using HPLC, DPPH assay, MTS assay, Griess reagent, and ELISA method. Our study found variations in three different varieties of *G. frondosa*-fermented wheat polysaccharides. Glucose was the predominant monosaccharide, followed by galactose and mannose. Each variety had a different molecular weight distribution, with GFA-wheat mainly present in fraction II, GFB-wheat in fraction I, and GFC-wheat in fraction III. At a concentration of 1.25 mg/mL, GFA-wheat and GFB-wheat polysaccharides increased DPPH scavenging ability by 76.8% and 58.7%, respectively. The polysaccharides showed no apparent toxic effect and enhanced the production of NO, IL-6, and TNF-*α* in RAW 246.7 macrophages. GFB-wheat polysaccharides demonstrated remarkable immunomodulatory properties at a concentration of 5 *μ*g/mL. Our study provides a theoretical basis for using *G. frondosa* in wheat staple agricultural products to improve human health.

## 1. Introduction

*Grifola frondosa* (*G. frondosa*) is a basidiomycete fungus that belongs to the family Grifolaceae and the order Polyporales (NCBI: txid5627). *G. frondosa*, known as Hui-Shu-Hua in Chinese, is a culinary medicinal mushroom in China [1]. *G. frondosa* is a traditional Chinese medicinal herb that has been used for centuries to improve energy levels, strengthen the immune system, and support respiratory and liver health [2]. Due to its strong umami flavor, it is also commonly used as a food ingredient or flavoring agent in human diets [3]. Over the last three decades, G. frondosa has been regarded as a healthy food and is famous worldwide because of its rich dietary fiber [4], protein, carbohydrates, vitamins, and minerals, as well as its low fat and calorie levels [5, 6]. Polysaccharides from *G. frondosa* also possess various pharmacological effects, such as antitumor and immunomodulatory effects [7–9], antioxidation [10], and antihyperglycemic effects [2]. Additionally, they can act effectively on the skin and hematopoietic stem cells [2, 11], providing significant benefits for human health.

It has been reported that *G. frondosa* fruiting bodies and mycelia contain an average of 33.53% and 47.84% carbohydrates on a dry weight basis, respectively [12, 13]. Therefore, producing only the mycelium for its bioactive polysaccharides instead of taking complicated steps to obtain polysaccharides from the *G. frondosa* fruiting body is a better choice [14, 15]. Solid-state fermentation (SSF) is an ideal method to meet the increasing demand for *G. frondosa* polysaccharides and their bioactive metabolites on a large scale and at a low cost [16, 17]. Several reports also stated that the SSF process could offer numerous advantages for both edible fungi and their solid chemical and enzyme substrates, enhancing the health properties of the final products. SSF of *Lentinus edodes* could enhance the total content of phenolic antioxidants [18]. The SSF of *Hericium erinaceus* could improve the nutritional value by increasing the protein amount, especially proteins rich in lysine and tryptophan [19].

Wheat (Triticum aestivum L.) has been the basic staple crop for the Chinese people for thousands of years. Countless daily Chinese foods, such as steamed buns, noodles, flatbreads, and dumplings, originate from wheat flour. Many studies have shown that a daily diet containing fermented cereals is beneficial for the prevention of free radical-mediated diseases [20]. Solid-state substrates such as whole grain cereals and the SSF process could increase their antioxidant potentials [17], enrich phenolic compounds, and improve sensory qualities [12, 21].

In previous studies, *G. frondosa* was inoculated into wheat [16], and the fermented products were tested for their taste characteristics and antioxidant activities [17, 22]. The results demonstrated that *G. frondosa*-fermented wheat contained more taste components and a higher antioxidant value than wheat. As a potential functional food, *G. frondosa*-fermented wheat flour can be produced at a lower cost and can be more conveniently incorporated into the human daily diet compared with the *G. frondosa* fruiting body, which would have broad prospects for human sanitation protection. However, there is still a knowledge gap regarding whether bioactive *G. frondosa* polysaccharides and their metabolites in the form of *G. frondosa*-fermented wheat flour provide antitumor and immunomodulatory activities. Furthermore, the differences in *G. frondosa* varieties may affect the structure and biological activities of polysaccharides, but previous studies have mainly focused on a single variety. Therefore, in this study, a systematic evaluation of the antitumor and immunomodulatory functions of *G. frondosa*-fermented wheat polysaccharides was performed in a model system of RAW 264.7 macrophages. At the same time, the differences in the solid fermentation products of different varieties of *G. frondosa* were also compared to reveal the differences between *G. frondosa*-fermented wheat products leading to functional differences. To the best of our knowledge, this is the first report on the immunomodulatory properties of SSF of wheat polysaccharides with different varieties of *G. frondosa*. This study provides basic theoretical support for the beneficial antioxidant and immunomodulatory properties of *G. frondosa*-fermented wheat flour, which could enhance wheat staple agricultural products and the popularization and application of *G. frondosa*.

## 2. Materials and Methods

### 2.1. Organism and Chemicals

The study used three different strains of *G. frondosa*, namely, *G. frondosa* A (strain: CGMCC 5.248), *G. frondosa* B (strain: CGMCC 5.702), and *G. frondosa* C (strain: ATCC 60891). They were obtained from China General Microbiological Culture Collection Center (CGMCC). *G. frondosa* A and *G. frondosa* B were local Chinese strains isolated from northern China, while *G. frondosa* C was isolated from Japan. RAW 264.7 macrophages were purchased from ATCC. Wheat grains of Malan No.1 variety were obtained from the local Chinese market and stored at room temperature. Whole grains that were not ground were used as solid substrates in the SSF process. Analytical-grade chemicals and solvents were used throughout the study.

### 2.2. Solid-State Fermentation

The method for *G. frondosa*-wheat SSF process was based on Chang's method with minor modifications [23]. The wheat grains were soaked with water, and according to the volume ratio, wheat grains to water of 1 : 1 were steamed for 40 min. After cooling, the cooked wheat grain was dispensed and compacted into 500 mL culture bottles. The filling height was 80% of the bottle height, and the bottles were autoclaved at 121°C for 90 min. All the *G. frondosa* strains were stored on slant PDA at 4°C and inoculated on PDA plates cultured at 25°C for 14 days. Until the PDA plates were full of active mycelia, 1 cm pieces of PDA plate were cut and transferred onto the surface of the autoclaved wheat grain culture. Each strain of *G. frondosa* was inoculated into three bottles. The wheat was then fermented for 30 days at 25°C in darkness and at a humidity of 70%. In addition, a blank control was prepared by following the same procedures as the *G. frondosa*-wheat fermented samples, but without the mycelium inoculation step. All the samples, including the *G. frondosa*-wheat fermented samples, wheat samples, and the *G. frondosa* active mycelia grown on PDA plate samples, were dried at 50°C in a vacuum drying oven for 6 hours. The dried samples were then ground using an electric grinder to obtain powdery samples.

### 2.3. Extraction of Polysaccharides

The method for extracting solubilized polysaccharides from the powdery samples was according to the previous method with minor modifications [24]. A total of 20 times the volume of deionized water was added to each powdery sample and stirred thoroughly. Then, the solutions were subjected to heat and reflux for extraction at 90°C for 4 hours. The samples were then centrifuged at 12,000 × *g* for 20 min at 4°C, and the supernatant fraction containing the solubilized polysaccharides was collected. The extraction process was repeated twice for precipitation, and the resulting supernatant fraction was precipitated with ethanol and deproteinized using 14% trichloroacetic acid. The resulting polysaccharides were dialyzed (MWCO 500 Da, Millipore) and lyophilized to obtain the final polysaccharide samples. The extraction yield was calculated as the weight of lyophilized powder per 100 g of original dry powder, and the total protein concentrations in the samples were determined using the Bradford method [25].

### 2.4. Determination of the Total Sugar Content, Monosaccharide Composition, and Mw Profile

The total sugar content of the lyophilized polysaccharide samples was determined by the phenol-sulfuric acid methods [26]. Monosaccharide composition was determined by high-performance liquid chromatography (HPLC) with an evaporative light scattering detector (6100 Chromachem, ESA Inc.) using a Waters 2695 system (Waters, Milford, MA, USA) following the procedure described by our previous study [27]. The molecular weight (Mw) profile of polysaccharides was determined by the gel permeation chromatography procedure involving the use of a Waters 2695 system (Waters, Milford, MA, USA) equipped with a TSK gel GMPW XL column (300 mm × 7.8 mm, Tosoh Corp., Tokyo, Japan). The polysaccharide samples were dissolved in deionized water at a concentration of 2 mg/mL and filtered through a 0.45 *μ*m filter before injection into the column. The column was eluted with 0.1 M NaNO_3_ at a flow rate of 0.5 mL/min, and the elution was monitored by a refractive index detector (2414, Waters). Pullulan standards were used to construct the calibration curve, and the molecular weight was determined using Waters Empower 3 software (Waters, Milford, MA, USA).

### 2.5. DPPH Radical Scavenging Assay

The percentage of DPPH scavenging activity for the polysaccharide samples was determined using the following equation: DPPH scavenging activity (%) = (1 − (As/Ab)) × 100%, where “As” denotes the absorbance after the reaction of the polysaccharides/ascorbic acid with DPPH and “Ab” refers to the absorbance value obtained when DPPH is reacted with distilled water. The measurements were performed in triplicate (*n* = 3). The methodology employed to assess the scavenging potential of the polysaccharide samples on DPPH-free radicals has been previously reported in a study by Zhai et al. [28].

### 2.6. Cell Culture

The RAW 264.7 macrophages (ATCC, Manassas VA) were cultured in DMEM medium supplemented with 10% FBS (Life Technologies) and 1% penicillin-streptomycin (GE Healthcare Life Sciences) and maintained in a humidified incubator at 37°C and 5% CO_2_.

### 2.7. Nitric Oxide (NO) Measurement

The measurement of NO was performed with minor modifications to previous study [29]. Cells were seeded at a density of 25,000 cells per well in a 96-well plate and allowed to grow for 24 hours. They were treated with different concentrations (ranging from 0.625 to 5 mg/mL) of sterile polysaccharides, with or without lipopolysaccharide (LPS), for a duration of 24 hours. Each concentration was tested in triplicate wells. The culture medium was considered as the normal control, whereas LPS was used as a positive control at a concentration of 1 *μ*g/mL. After treatment, the supernatant was collected and the nitric oxide (NO) levels were quantified using the Griess assay in a 96-well plate. To measure NO, 100 *μ*L of the supernatant or varying concentrations of sodium nitrite as a standard were added to a 96-well plate. Next, 100 *μ*L of Griess reagent was added to the samples and allowed to incubate at room temperature for 5 minutes. The absorbance was then measured at 550 nm using a Synergy H1 microplate reader (BioTek, USA), and the quantity of NO generated was calculated by comparing the results with a sodium nitrite standard curve (*y* = 0.0102*x* + 0.0529, *R*^2^ = 1, where “*y*” represents the absorbance value and “*x*” represents the NO concentration in *μ*m). All measurements were performed in triplicate (*n* = 3).

### 2.8. The Level of Interleukin- (IL-) 6 and Tumor Necrosis Factor-*α* (TNF-*α*)

To quantify the production of IL-6 and TNF-*α* in the supernatant of the treated cells, an enzyme-linked immunosorbent assay (ELISA) was performed using ELISA Max™ Deluxe kits (BioLegend, San Diego, CA, USA), following the manufacturer's instructions. The absorbance was measured at 450 nm using a Synergy H1 microplate reader (BioTek, USA). The concentration of IL-6 and TNF-*α* was determined by comparing with standard curves obtained using recombinant mouse IL-6 and TNF-*α* standards. All measurements were performed in triplicate (*n* = 3).

### 2.9. Measurement of Cell Proliferation

The MTS assay is a colorimetric method that measures the number of viable cells in a culture. It is based on the ability of viable cells to reduce MTS (3-(4,5-dimethylthiazol-2-yl)-5-(3-carboxymethoxyphenyl)-2-(4-sulfophenyl)-2H-tetrazolium) to formazan, a colored compound that can be measured spectrophotometrically. In this assay, after the collection of the culture supernatant for NO determination, the cells were incubated with MTS reagent for 3 hours, and the absorbance was measured at 490 nm [30]. The amount of formazan produced is proportional to the number of viable cells in the culture.

### 2.10. Statistical Analysis

Statistical analysis was performed using SPSS 26.0 software (IBM, Armonk, NY, USA). All experiments were carried out with at least three independent replicates, and the results are presented as mean ± standard deviation (SD). One-way ANOVA with Duncan's test was used to assess statistical significance, and differences with a *P* value less than 0.05 were considered significant.

## 3. Results

### 3.1. Analysis of the Physicochemical Properties of Polysaccharides

The water-soluble polysaccharide samples were extracted from seven crude polysaccharide samples obtained from three varieties of *G. frondosa*, namely, *G. frondosa* A (GFA), *G. frondosa* B (GFB), and *G. frondosa* C (GFC), which were cultured in PDA plates or by wheat solid-state fermentation. The extraction was carried out by hot water extraction. The physicochemical properties of these polysaccharide samples are presented in Table 1.

The total yield of *G. frondosa* mycelium polysaccharide was significantly higher when cultured with fermented wheat compared to PDA plates, with that cultured on PDA plates being 3.54%-5.78%, and that cultured with fermented wheat being 22.46%-25.33%. It could be attributed to the availability of nutrients and favorable conditions provided by the wheat substrate for fungal growth. There were no significant differences between different strains of *G. frondosa* in the total yield of polysaccharides. The total sugar contents of all the samples were similar, in the range of 62.97%-72.65%, which suggested that the extraction process was effective in isolating the polysaccharides from the fungal mycelium, and there were no significant differences in the purity of the polysaccharides among the samples. The low protein contents (0.12%-0.45%) of the samples indicated that the extraction method used was effective in removing protein impurities.

Five types of monosaccharides (mannose, glucose, galactose, rhamnose, and arabinose) were detected in all the samples. In the group cultured in PDA plates, the molar ratios of monosaccharides were Man : Glc : Gal : Rha : Ara = 1.00 : (12.02 − 15.34): (0.87 − 1.43): (0.32 − 0.98): (0.12 − 0.92). In the wheat SSF group, the molar ratios were Man : Glc : Gal : Rha : Ara = 1.00 : (21.45 − 22.45): (2.23 − 3.78): (0.38 − 0.65): (0.12 − 0.43). There were significant differences in the molar ratios of monosaccharide composition between the polysaccharide samples cultured in PDA plates and wheat SSF. The glucose content was significantly affected by the wheat substrate, and the samples containing wheat had a high glucose content. In the wheat SSF group, the galactose contents were significantly increased, especially in the sample of GFC-fermented wheat, which was 4.3 times that on PDA plates and 3.1 times that of wheat. The presence of rhamnose and arabinose in both groups of samples was relatively low, but with higher proportions in the wheat SSF group. Overall, the results suggested that the monosaccharide composition of polysaccharide samples was significantly influenced by the culture conditions, particularly the substrate used for fermentation.

### 3.2. The Mw Profile Changes in Polysaccharides

**Figure 1:**
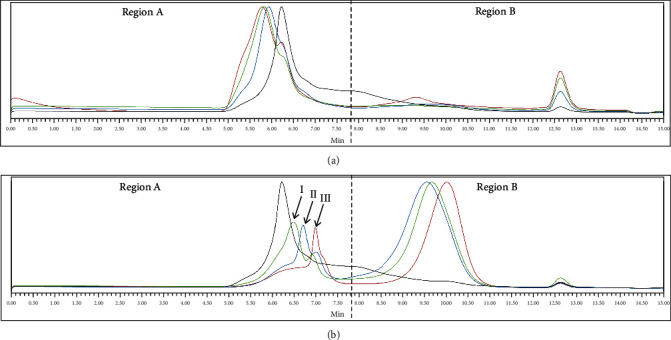
HPLC analysis of Mw distribution of the G. frondosa-wheat polysaccharides: (a) HPLC chromatogram of *G. frondosa* mycelium on PDA plates and (b) HPLC chromatogram of *G. frondosa*-fermented wheat mycelium. Different colors indicate different polysaccharide fractions: wheat is displayed in black, GFA is displayed in blue, GFB is displayed in green, and GFC is displayed in red. The chromatogram is divided into two regions, with a retention time of 7.8 minutes and a corresponding Mw of 560 kDa as the boundary. The Mw distributed in region A is greater than 560 kDa, while the Mw distributed in region B is less than 560 kDa.

The HPLC profile shown in Figure 1 displays the Mw distribution of the polysaccharide samples extracted from the three varieties of G. frondosa and wheat. The Mw distributions of the polysaccharides extracted from G. frondosa were found to be very different from each other and wheat. The wheat sample (shown as a black line) was used as a control, and it exhibited only one main fraction with a retention time of 6.225 min, representing an Mw of approximately 1.77 × 10^4^ kDa.

Figure 1(a) shows that the PDA-cultured mycelium of the three *G. frondosa* varieties also had one main fraction each, which had a shorter retention time than that of wheat, indicating that their Mw was higher than that of wheat. Among them, GFC mycelium had the shortest retention time, followed by GFB and GFA, indicating that their polysaccharide Mw is GFC > GFB > GFA, where GFA was estimated to be 3.36 × 10^4^ kDa, GFB was estimated to be 4.48 × 10^4^ kDa, and GFC was estimated to be 5.00 × 10^4^ kDa.

Figure 1(b) shows that the Mw profile of *G. frondosa*-wheat polysaccharides had significant differences compared to Figure 1(a), indicating a decrease in Mw after the SSF process. The higher Mw region (region A) exhibited three main fractions, with fraction I having a Mw of 9.36 × 10^3^ kDa, fraction II having a Mw of 6.08 × 10^3^ kDa, and fraction III having a Mw of 3.30 × 10^3^ kDa. The Mw profile of GFA-wheat was mainly distributed in fraction II, followed by fraction III; GFB-wheat was mainly distributed in fraction I and fraction III; and GFC-wheat was mainly distributed in fraction III. In the lower Mw region B, each of the three *G. frondosa* varieties had a large main fraction. The fraction of GFA-wheat had the largest Mw, followed by GFB-wheat and GFC-wheat.

Overall, the polysaccharide composition of *G. frondosa*-fermented wheat obtained from the three different varieties of *G. frondosa* was significantly different. After the SSF process, the polysaccharide components underwent degradation, and the trend of shifting to the lower Mw region was obvious.

### 3.3. Antioxidant Activity of G. frondosa-Fermented Wheat Polysaccharides

**Figure 2:**
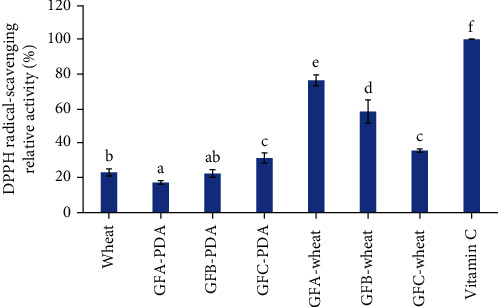
DPPH radical scavenging relative activity of different polysaccharide samples from *G. frondosa*, wheat, and their SSF (solid-state fermentation) products. Samples were run in triplicate, and the results from one representative experiment are shown (*n* = 3). The relative activity of DPPH radical scavenging is shown on the *y*-axis, and the different samples are labeled on the *x*-axis. Vitamin C was used as a positive control, with its relative activity set at 100%. The figure could help to compare the antioxidant activity of the different samples and evaluate the effectiveness of SSF in enhancing their antioxidant properties.

The results from the DPPH scavenging assay indicated that the polysaccharides obtained from G. frondosa-fermented wheat have increased antioxidant activity compared to those from G. frondosa mycelium and wheat alone.  Figure 2 illustrates that GFA-wheat and GFB-wheat polysaccharides exhibited the highest increase in DPPH scavenging ability, with values of 59.3% and 36.0%, respectively, compared to their G. frondosa mycelium counterparts. However, there was little difference in the DPPH scavenging activity of GFC before and after the SSF process. According to their HPLC profiles, fractions I and II of the polysaccharide components may have a higher DPPH scavenging ability than fraction III. Overall, these results suggested that the SSF process might have a significant impact on the antioxidant activity of *G. frondosa* polysaccharides, with GFA-wheat and GFB-wheat polysaccharides exhibiting the most promising antioxidant potential.

### 3.4. The Effects of Polysaccharides of G. frondosa-Fermented Wheat on Immunomodulatory Activities

#### 3.4.1. Cell Viability

**Figure 3:**
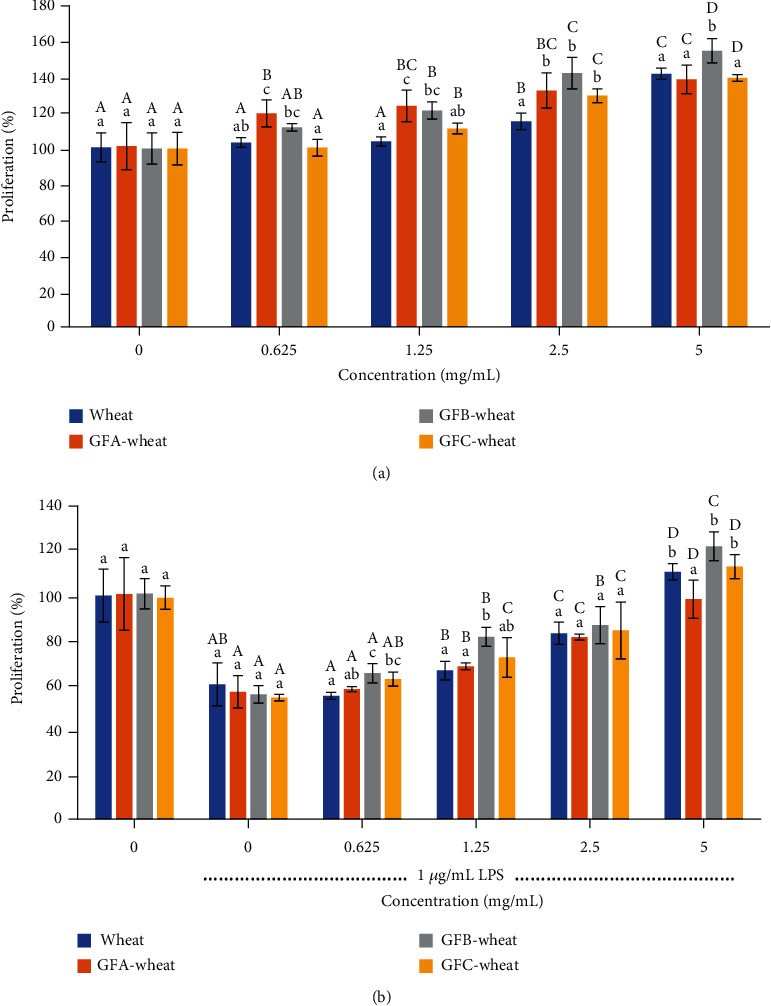
Effects of the polysaccharide samples of *G. frondosa*-fermented wheat on the proliferation generated by RAW 264.7 cells with or without stimulation by LPS: (a) RAW 264.7 cells without LPS and (b) RAW264.7 cells treated with 1 *μ*g/mL LPS. The *x*-axis shows the concentration of the polysaccharide samples, while the *y*-axis shows the cell proliferation rate expressed as a percentage relative to the control group. Samples were run in triplicate, and the results from one representative experiment are shown (*n* = 3). Columns at the same concentration with different lowercase letters are significantly different (*P* < 0.05). Columns representing the same sample (same color) with different capital letters are significantly different (*P* < 0.05). Overall, the results demonstrate that the *G. frondosa*-fermented wheat polysaccharides can effectively promote the proliferation of RAW 264.7 cells with or without LPS stimulation.

The MTS assay is a commonly used method to evaluate cell viability and proliferation. In this study, the assay was used to evaluate the effect of polysaccharide samples on both normal RAW 264.7 cells and cells treated with LPS, which induces inflammation in the cells.  Figure 3(a) shows that all polysaccharide samples obtained from wheat or *G. frondosa*-fermented wheat increased the proliferation of normal RAW 264.7 cells within the concentration range of 0.625-5 mg/mL without causing cytotoxicity. Among the samples, GFB-wheat showed the highest proliferation effect on cells, reaching up to 154%.  Figure 3(b) shows that, when the same polysaccharide samples were tested on LPS-treated RAW 264.7 cells, the cell viability was significantly reduced by 50%. However, adding polysaccharide samples to these cells promoted cell proliferation and gradually recovered cell viability as the concentration increased. The recovery ability of GFB-wheat was the highest, reaching 122%. The results suggested that the polysaccharide samples from wheat and *G. frondosa*-fermented wheat could promote the proliferation of immune cells without causing cytotoxicity. Moreover, it is worth noting that the polysaccharide sample of GFB-wheat has a significant promotion and recovery effect on cell proliferation, indicating that this polysaccharide component plays an important role in the activation and protection of immune cells.

#### 3.4.2. NO Production

**Figure 4:**
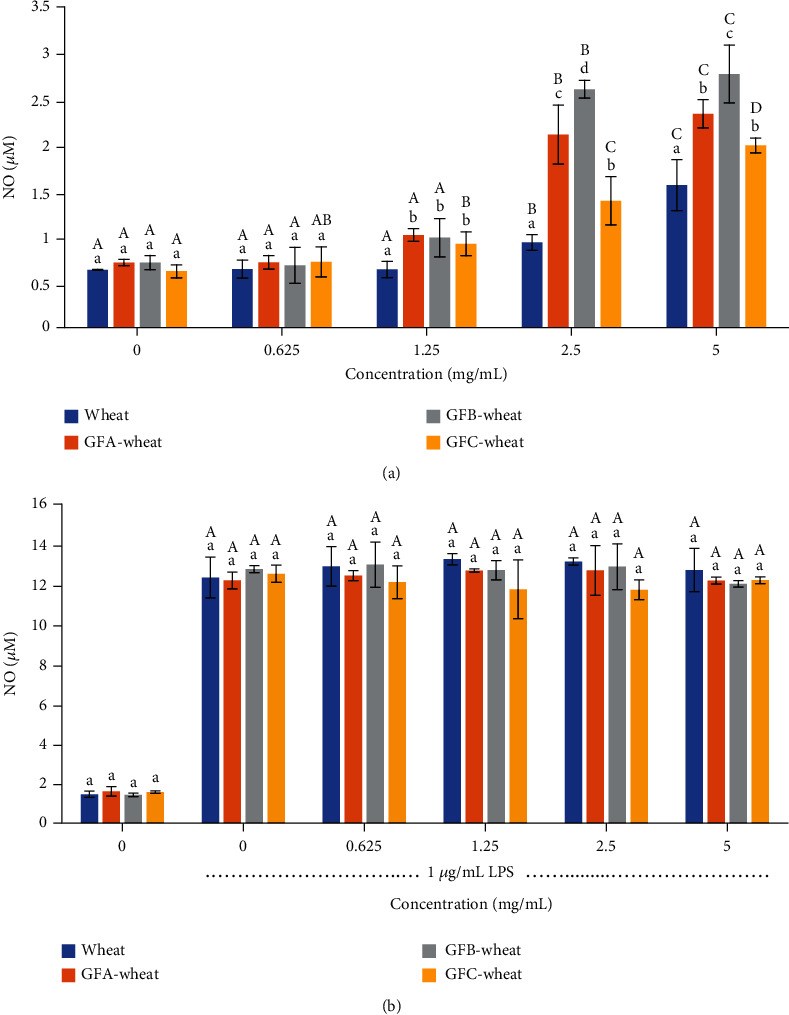
Induction of nitric oxide (NO) release in RAW 264.7 cells treated with polysaccharide samples of *G. frondosa*-fermented wheat: (a) RAW 264.7 cells without LPS and (b) RAW264.7 cells treated with 1 *μ*g/mL LPS. Samples were run in triplicate, and the results from one representative experiment are shown (*n* = 3). Columns at the same concentration with different lowercase letters are significantly different (*P* < 0.05). Columns representing the same sample (same color) with different capital letters are significantly different (*P* < 0.05).

The results from Figure 4 indicated that the polysaccharides from *G. frondosa*-fermented wheat have a significant effect on promoting the synthesis of NO in RAW 264.7 cells, which is an important regulator of immune function. The increase in NO production was concentration-dependent and all samples promoted NO synthesis, with GFB-wheat showing the most significant effect, up to 2.76 *μ*m, which was 3.88 times that produced in the initial state, followed by GFA-wheat and GFC-wheat. These findings suggested that the polysaccharides from *G. frondosa*-fermented wheat can enhance cellular immunity by promoting NO synthesis. However, none of the polysaccharide samples significantly promoted or inhibited NO synthesis in LPS-treated inflammatory cells (Figure 4(b)), indicating that these polysaccharides do not exacerbate inflammation or stimulate further inflammation. Overall, these results suggested that the polysaccharides from *G. frondosa*-fermented wheat might have the potential as immunomodulatory agents.

#### 3.4.3. The Cytokines TNF-*α* and IL-6

**Figure 5:**
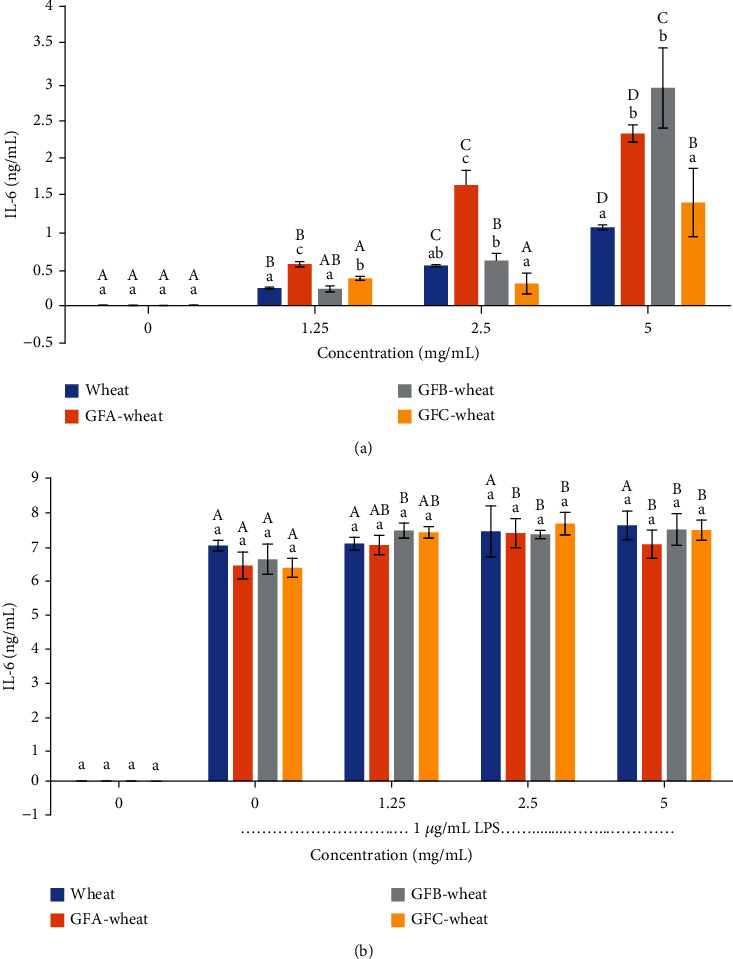
Induction of IL-6 release in RAW 264.7 cells treated with polysaccharide samples of *G. frondosa*-fermented wheat: (a) RAW 264.7 cells without LPS and (b) RAW264.7 cells treated with 1 *μ*g/mL LPS. Samples were run in triplicate, and the results from one representative experiment are shown (*n* = 3). Columns at the same concentration with different lowercase letters are significantly different (*P* < 0.05). Columns representing the same sample (same color) with different capital letters are significantly different (*P* < 0.05).

**Figure 6:**
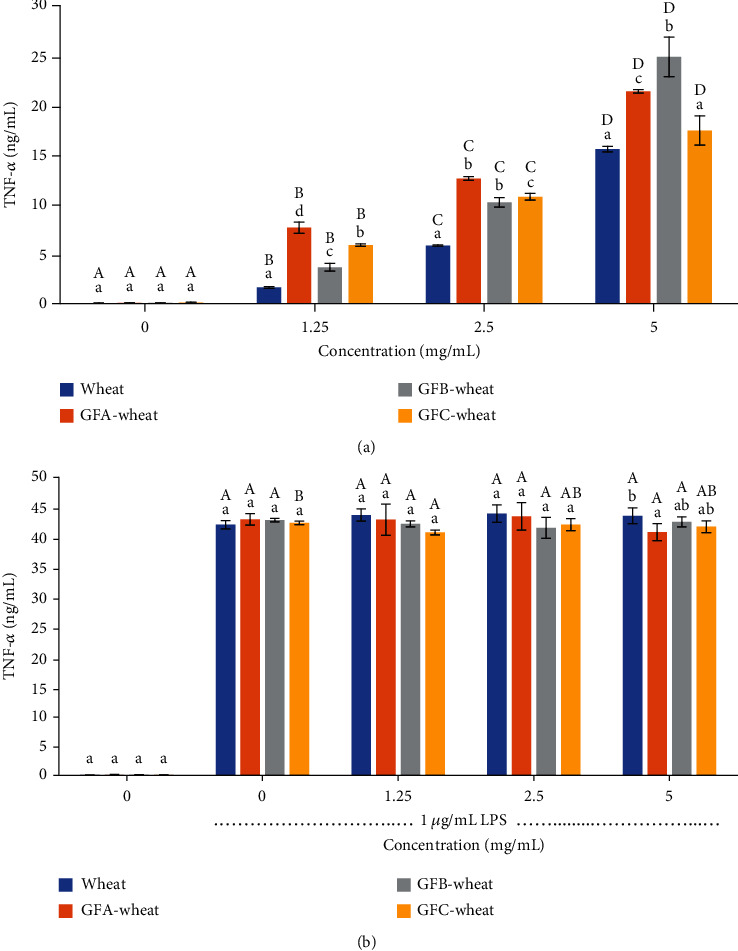
Induction of TNF-*α* release in RAW 264.7 cells treated with polysaccharide samples of *G. frondosa*-fermented wheat: (a) RAW 264.7 cells without LPS treatment and (b) RAW264.7 cells treated with 1 *μ*g/mL LPS. Samples were run in triplicate, and the results from one representative experiment are shown (*n* = 3). Columns at the same concentration with different lowercase letters are significantly different (*P* < 0.05). Columns representing the same sample (same color) with different capital letters are significantly different (*P* < 0.05).

The ELISA was used to determine the content of IL-6 (Figure 5) and TNF-*α* (Figure 6) in the cell culture medium. As shown in Figures 5 and 6, the results suggested that the polysaccharides from *G. frondosa*-fermented wheat can promote the secretion of proinflammatory cytokines, but far from the level needed to cause cell inflammation, indicating that they may play a role in enhancing the immune response of macrophages. Additionally, the polysaccharide from GFB-wheat had the most significant promotion effect, with IL-6 up to 2.91 ng/mL and TNF up to 24.84 ng/mL, indicating that it may be a potential candidate for the development of immunomodulatory agents.

However, it is important to note that further studies are needed to investigate the underlying mechanisms by which these polysaccharides exert their immunomodulatory effects.

## 4. Discussion

The hot water extraction method has been widely used to extract bioactive polysaccharides from *G. frondosa*, and it was first employed in the 1980s [31]. This method has also been used to extract polysaccharides from fungal-fermented grains in many studies [32, 33]. In this study, we used this method to extract polysaccharides from *G. frondosa* mycelium and polysaccharides from *G. frondosa*-fermented wheat products. The crude polysaccharide total yield in the mycelium was 3.54-5.78 g/100 g, which is comparable to other studies of *G. frondosa* mycelium that have reported a total yield of approximately 7.4-8.7 g/100 g under similar extraction and detection methods [12, 34]. The variations in total yield among different *G. frondosa* samples may be due to factors such as the variety, cultivation period, and cultivation environment. In our study, the total yield for the *G. frondosa*-fermented wheat groups was 22.46%-25.33%, which was much higher than the yield of 15.64% reported for *Hericium erinaceus*-fermented wheat products [32].

*G. frondosa* mycelium polysaccharides have been reported to contain eight monosaccharides, namely, glucose, mannose, galactose, xylose, arabinose, fucose, rhamnose, and ribose [35–37]. In our study, only five types of monosaccharides, mannose, glucose, galactose, rhamnose, and arabinose were detected. It is worth noting that in our study, the solid fermentation samples were extracted together with the wheat substrate, so the glucose content was significantly higher than that in the pure mycelium samples. In addition, the contents of galactose and rhamnose in the *G. frondosa*-fermented wheat groups were significantly higher than those in the wheat control group. This result also indicated that because of the SSF process in *G. frondosa*, the starch in wheat is converted into functional polysaccharides [17, 19]. We found that the composition of polysaccharides produced by fermentation of different varieties of *G. frondosa* was different; the special higher monosaccharide content was rhamnose for *G. frondosa* A, mannose for *G. frondosa* B, and galactose and arabinose for *G. frondosa* C. Therefore, it could be concluded that the polysaccharide structures obtained by different varieties of *G. frondosa*-fermented wheat are different and, thus, reflect different immune activities.

The Mw of water-soluble polysaccharides of *G. frondosa* mycelium shows a diverse distribution. According to Su's research, two major macromolecular populations of *G. frondosa* polysaccharides were 722.7 kDa and 19.6 kDa [38]. Moreover, Ma's studies have shown that the average Mw of *G. frondosa* polysaccharide is 889 kDa [39]. Our study showed that the Mw range of the polysaccharides extracted from different varieties of *G. frondosa* mycelium samples was 3.36 − 5.00 × 10^4^ kDa. These differences in Mw could be attributed to various factors such as the type of *G. frondosa* variety, extraction methods, and the presence of different hydrolyzing enzymes during the SSF process [17].

In addition to the Mw, the composition and distribution of polysaccharide fractions also vary among different *G. frondosa* varieties and SSF processes. Our study showed that, although GFA, GFB, and GFC were the varieties that all belong to *G. frondosa*, it was obvious that after the SSF process with the same type of wheat, GFA-wheat was mainly distributed in fraction II, followed by fraction III; GFB-wheat was mainly distributed in fraction I and fraction III; and GFC-wheat was mainly distributed in fraction III. This might be related to the levels of the types and quantities of hydrolyzing enzymes released by the different varieties of *G. frondosa* in the process of SSF [20, 21]. Moreover, the action of enzymes, such as pectinases, cellulases, *α*-amylases, xylanase, *β*-glucosidase, *β*-xylosidase, *β*-galactosidase, and *β*-hesperidinase, also plays an important role in the mobilization of bioactive phenolic compounds during SSF [40], and it is very likely that new types of polysaccharides are produced during the fermentation process due to the metabolism of *G. frondosa* fungus [16, 41]. This also explains why in our study, although all three types of *G. frondosa*-fermented wheat polysaccharides had antioxidant and immune activation effects, their activities were significantly different. In the final analysis, there was a difference in polysaccharide structure. Therefore, further in-depth analysis of the composition and structure of these polysaccharides is needed to elucidate the polysaccharide structure related to certain functions. This knowledge will provide a better understanding of the health-promoting effects of *G. frondosa* polysaccharides and can be utilized in the development of functional foods and supplements.

Replenishing antioxidants in the diet can reduce oxidative damage and delay aging, which is beneficial for protecting human health [18, 20]. According to Bhanja Dey's research, *Rhizopus oryzae*-fermented wheat products showed a higher DPPH radical scavenging activity than the wheat control [21]. Our present study showed that the relative DPPH radical scavenging activity of the polysaccharides from wheat itself or the *G. frondosa* mycelium was low; however, the polysaccharides from the GFA- and GFB-wheat samples exhibited strong DPPH radical scavenging activity. At 1.25 mg/mL, the scavenging rates of DPPH for GFA-wheat and GFB-wheat were 76.8% and 58.7%, respectively.

Previous studies have reported that the antioxidant properties of polysaccharides are associated with factors such as the composition of monosaccharides, molecular weight (Mw), and types of glycosidic bonds [42]. Moreover, the Mw of polysaccharides is known to have a significant impact on their antioxidant activity, as lower Mw polysaccharides tend to exhibit stronger antioxidant properties, including better scavenging of DPPH radicals [43, 44]. In Chen's research, the purified *G. frondosa* polysaccharide fractions of GFP-1, GFP-2, and GFP-3 at 3.0 mg/mL were 49%, 78%, and 66%, respectively [45]. Another *G. frondosa* polysaccharide fraction of Se-GFP-22, at 1 mg/mL, had a 46% scavenging rate [46]. In our study, the polysaccharides from different varieties of *G. frondosa* showed different DPPH scavenging activities. Moreover, the polysaccharides from the *G. frondosa*-fermented wheat samples with a lower Mw were found to have higher antioxidant activities. After comparison, the polysaccharides from GFA-wheat and GFB-wheat were found to have similar DPPH radical scavenging activities as purified polysaccharide fractions of *G. frondos*a reported in other studies. These findings suggest that GFA and GFB strains of *G. frondosa* are good strains for use in the SSF of wheat to enhance antioxidant activity.

The proliferation results in our study showed that the polysaccharide samples from G. frondosa, wheat, and *G. frondosa*-fermented wheat not only did not show any toxicity but also promoted macrophage proliferation with or without LPS. This finding suggests that these polysaccharides may have immunomodulatory effects and could be used as functional foods to enhance immune function. Previous studies have also reported the positive effects of pure polysaccharides from *G. frondosa* on macrophage proliferation [7, 39]. The results of our study further support the potential of *G. frondosa*-fermented wheat polysaccharides as a functional food for daily diets.

Immunomodulation is the most well-known effect of *G. frondosa* polysaccharides and has been confirmed by numerous studies [2, 35, 39]. The levels of NO and proinflammatory cytokines such as IL-6 and TNF-*α* are commonly used as indicators of immunomodulation and inflammation. In our study, we found that all *G. frondosa*-fermented wheat polysaccharides could enhance the production of NO, IL-6, and TNF-*α* in a dose-dependent manner, exhibiting immune-modulatory properties. GFA-wheat at 5 *μ*g/mL maximally increased the production of NO, IL-6, and TNF-*α* by 1.5-, 2.2-, and 1.37-fold, respectively, compared with the wheat control group. The values for 5 *μ*g/mL GFB-wheat were 1.77-, 2.78-, and 1.59-fold, respectively. The values for 5 *μ*g/mL GFC-wheat were 1.27-, 1.31-, and 1.12-fold, respectively. Among them, GFB-wheat demonstrated the highest immune-modulatory activity. According to Mao's study, a water-soluble *G. frondosa* polysaccharide GP11 at 1 mg/mL could maximally increase the production of NO by 7.2-fold and TNF-*α* by 1.7-fold compared with the normal control group [35]. A neutral polysaccharide from the fruiting body of G. frondosa (GFP-A) at 4 mg/mL could increase the production of TNF-*α* by 2.3-fold [7]. These results indicated that compared with wheat polysaccharides and other purified *G. frondosa* polysaccharides, the *G. frondosa*-fermented wheat polysaccharides in our study could effectively induce the functional activation of RAW264.7 cells.

Our study also assessed the anti-inflammatory properties of polysaccharides from *G. frondosa*-fermented wheat in LPS-stimulated RAW 264.7 cells. We found that *G. frondosa*-fermented wheat polysaccharides not only promote the secretion of inflammatory factors by macrophages but also do not aggravate the inflammatory response of inflammatory cells. This result demonstrates that it can safely and effectively activate the immune response of macrophages without causing cell inflammation and damage and is a good immune activator. However, it is worth noting that the anti-inflammatory activity of *G. frondosa*-fermented wheat polysaccharides observed in this study is likely due to their ability to modulate the immune response rather than directly inhibiting inflammation. Therefore, further studies are needed to fully elucidate the mechanisms underlying the anti-inflammatory effects of *G. frondosa*-fermented wheat polysaccharides. It also should be noted that while the production of NO, IL-6, and TNF-*α* is indicative of immune-modulation and inflammation, excessive production of these cytokines can also lead to detrimental effects on human health. Therefore, further studies are needed to carefully evaluate the dosage and duration of intake of *G. frondosa*-fermented wheat polysaccharides as a functional food for daily diets to avoid potential adverse effects.

## 5. Conclusion

In the present study, the combination of the culinary medicinal fungus *G. frondosa* and the Chinese staple food wheat in fermented biologically active substances has the potential to provide a functional food with immune-modulatory effects that can be easily integrated into daily diets. In contrast to the polysaccharides extracted from the pure *G. frondosa* fruiting body or mycelia, the polysaccharides extracted from the three varieties of *G. frondosa*-fermented wheat flour in our study could also promote the proliferation of RAW 264.7 macrophages and exert outstanding immune-modulatory effects through NO, TNF-*α*, and IL-6 induction. In addition, compared with obtaining pure *G. frondosa* polysaccharides, the use of *G. frondosa*-fermented wheat flour can simplify the process and lower the cost of production, making it feasible for large-scale preparation. The study provides a theoretical basis for using G. frondosa in wheat staple agricultural products to improve human health.

## Figures and Tables

**Table 1:** Physicochemical properties of *G. frondosa* mycelium and fermented wheat polysaccharide samples.

Sample	% (w/w)			Molar ratios of monosaccharide composition^C^				
	Yield (g/100 g)	Total sugar^A^	Protein^B^	Man	Glc	Gal	Rha	Ara
Wheat	26.53 ± 6.62^b^	72.65 ± 8.74^a^	0.45 ± 0.06^c^	1.00	26.45^c^	1.23^a^	0.15^a^	0.35^b^
GFA-PDA	3.54 ± 0.26^a^	67.43 ± 6.32^a^	0.12 ± 0.07^a^	1.00	15.34^ab^	1.13^a^	0.67^d^	0.92^d^
GFB-PDA	5.78 ± 0.83^a^	62.97 ± 4.89^a^	0.22 ± 0.02^ab^	1.00	13.98^a^	1.43^a^	0.32^b^	0.12^a^
GFC-PDA	4.47 ± 0.65^a^	70.37 ± 8.62^a^	0.18 ± 0.04^ab^	1.00	12.02^a^	0.87^a^	0.98^e^	0.75^c^
GFA-wheat	25.33 ± 3.25^b^	72.34 ± 7.55^a^	0.23 ± 0.08^b^	1.00	22.34^c^	2.43^b^	0.65^d^	0.12^a^
GFB-wheat	22.46 ± 5.89^b^	64.86 ± 3.69^a^	0.35 ± 0.07^c^	1.00	21.45^bc^	2.23^b^	0.38^bc^	0.14^a^
GFC-wheat	24.62 ± 7.16^b^	69.02 ± 4.91^a^	0.42 ± 0.03^c^	1.00	22.45^bc^	3.78^c^	0.45^c^	0.43^b^

^A^Polysaccharide content was determined by subtracting free reducing sugars from total reducing sugars after hydrolysis with H_2_SO_4_ as described in Materials and Methods. Each value is expressed as mean ± SD (*n* = 3). Means with different letters within a line are significantly different (*P* < 0.05). ^B^Protein content was determined by the Bradford method. Each value is expressed as mean ± SD (*n* = 3). Means with different letters within a line are significantly different (*P* < 0.05). ^C^Man: mannose; Glc: glucose; Gal: galactose; Rha: rhamnose; Ara: arabinose. Each value is expressed as a mean ratio of mannose content. Means with different letters within a line are significantly different (*P* < 0.05).

## Data Availability

All data generated or analyzed during this study are included in this published article.

## Abbreviations

Ara: Arabinose
CGMCC: China General Microbiological Culture Collection Center
DPPH: 2,2-Diphenyl-1-picrylhydrazyl
ELISA: Enzyme-linked immunosorbent assay
Gal: Galactose
GFA: *Grifola frondosa* A
GFB: *Grifola frondosa* B
GFC: *Grifola frondosa* C
Glc: Glucose
HPLC: High-performance liquid chromatography
IL: Interleukin
LPS: Lipopolysaccharide
Man: Mannose
MTS: (3-(4,5-Dimethylthiazol-2-yl)-5-(3-carboxymethoxyphenyl)-2-(4-sulfophenyl)-2H-tetrazolium)
Mw: Molecular weight
NO: Nitric oxide
RID: Refractive index detector
Rha: Rhamnose
SD: Standard deviation
SSF: Solid-state fermentation
TCA: Trichloroacetic acid
TNF-*α*: Tumor necrosis factor-*α*.
